# Supplementary material for: A machine learning-based chemoproteomic approach to identify drug targets and binding sites in complex proteomes
Source: Nat Commun. 2020 Aug 21;11:4200. doi: 10.1038/s41467-020-18071-x (PMC7442650; doi:10.1038/s41467-020-18071-x)
Supplement: Supplementary file 1 — Supplementary Information [file 41467_2020_18071_MOESM1_ESM.pdf]

A machine learning-based chemoproteomic approach to identify drug targets and binding sites in complex proteomes

Piazza and Beaton et al., 2020

Supplementary Information

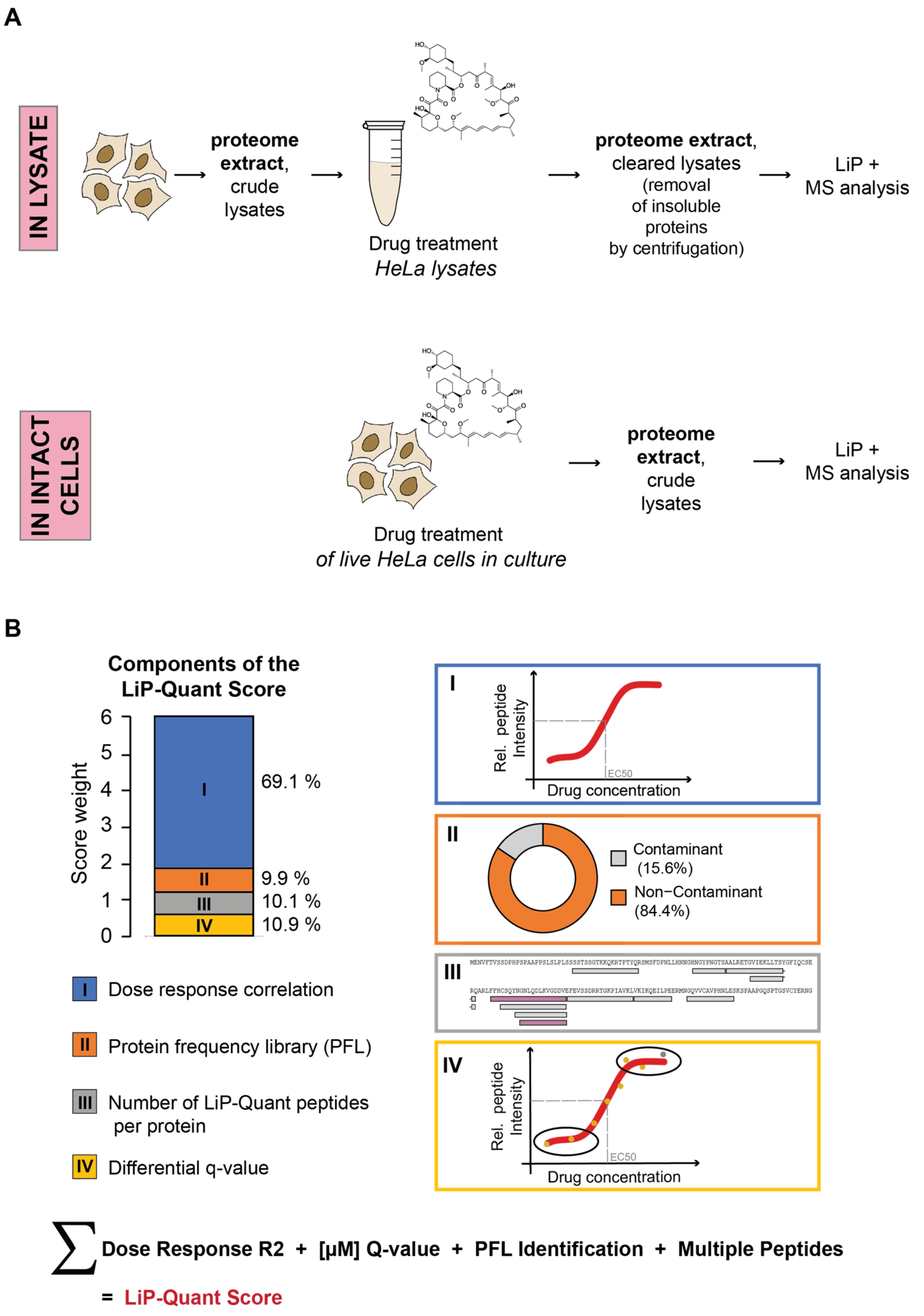

Supplementary Figure 1: Machine learning-derived discriminators for drug target identification in LiP-Quant experiments

A: Experimental procedure for the two kinds of LiP assays described in the main text: treatment of HeLa cell lysates (top), and the direct treatment of live HeLa cells in culture prior to lysis (bottom). **B:** The LiP-Quant score is based on 4 components including the correlation coefficient to a sigmoidal trend of the isothermal dose-response profile (I), the protein's frequency as a common contaminant in LiP-Quant experiments (Protein Frequency Library, PFL) (II), the number of LiP-Quant peptides assigned per protein (III) and the statistical significance of the relative peptide abundance between two points (IV). The stacked histogram shows the machine learning-derived relative weight of each component expressed as percentage of the LiP-Quant score over a possible maximum score of 6.

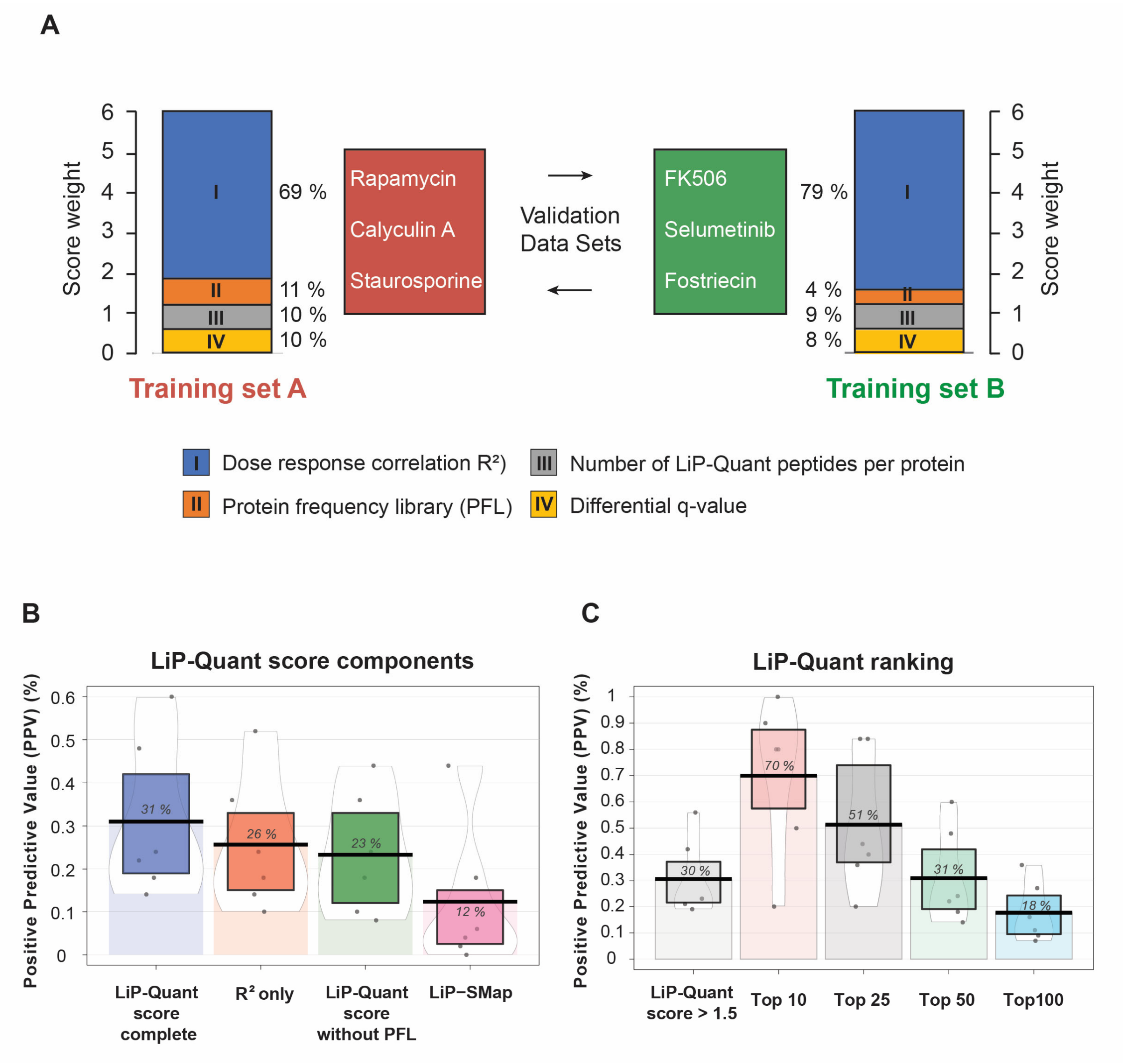

Supplementary Figure 2: LiP-Quant data analysis pipeline, its development, validation and machine learning training

A: Schematic of ground truth experiments used to determine sub-score weightings contributing to the LiP-Quant score. Two independent data sets, each from three experiments using three drugs with known targets, were analyzed by linear discriminant analysis to determine parameters that contribute to positive target identification. The weightings derived from each of these training sets was then validated on an independent data set, again each consisting of three experiments using three different drugs with known targets (i.e. trained on A and tested on B and vice versa). Four parameters were identified that contribute to positive target identification for each training set. The criteria include the correlation coefficient (R^2) to a sigmoidal trend of the isothermal drug dose-response profile, whether the identified protein is a common contaminant in LiP-Quant experiments (Protein Frequency Library (PFL), the number of LiP-Quant peptides per protein and the statistical significance of the relative abundance of a LiP peptide between drug and vehicle treated samples. The weightings of these parameters were found to be very stable. **B:** Positive Predictive Value (PPV) or precision of 3 different protein-small molecule interaction predictors: LiP-Quant using all 4 machine learning-derived components (LiP-Quant score complete) (Supplementary Figure 1), peptides ranked exclusively by the dose response correlation parameter (R^2 only), LiP-Quant excluding the common contaminant filtering (LiP-Quant score without PFL) (Supplementary Figure 1), and LiP-SMap [13]. Boxes show the interval of the interquartile range and bean plots show the

smoothed density curve relative to the full data distributions. The horizontal bars represent the means of the number of true positives peptides found in the experiments shown in this work. **C:** Positive predictive value calculated as in panel B when considering as positive hits the peptides with LiP-Quant score > 1.5, or the top 10, 25, 50 and 100 ranking peptides. Peptides scoring > 1.5 are generally contained in the top 50 scoring peptides. The positive predictive value and data representation calculations are the same as in panel B.

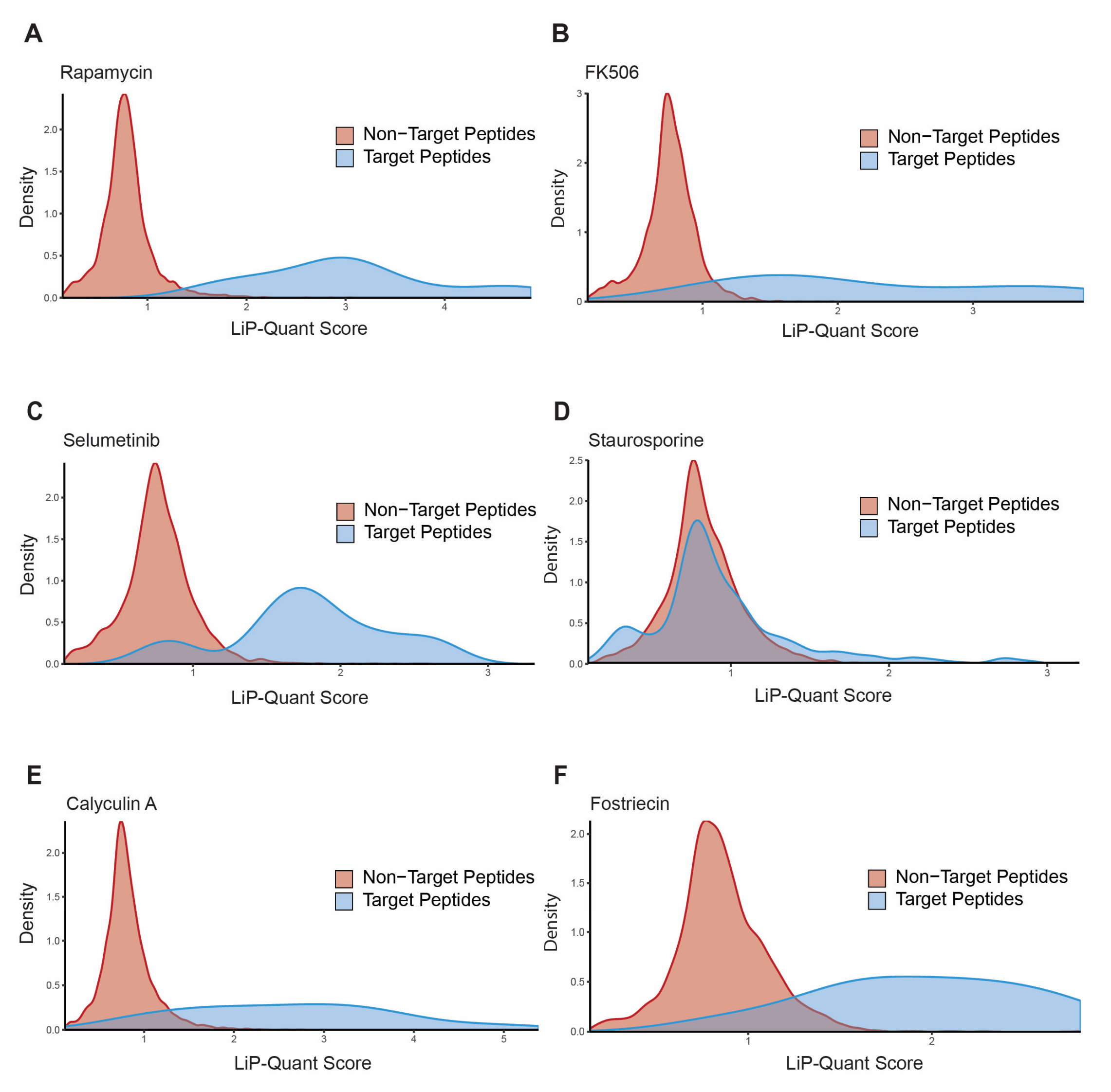

Supplementary Figure 3: Density plots of LiP-Quant score distributions

A – F: The distribution of LiP-Quant scores (Gaussian smoothed kernel density) for each LiP-Quant HeLa experiment. The staurosporine target density plot is larger and shifted to the left due to promiscuity of the compound and the extremely high number of potential targets (the human kinome: n = 512) that cannot be exhaustively captured by a single LiP experiment (excluded from LiP score threshold calculation for this reason).

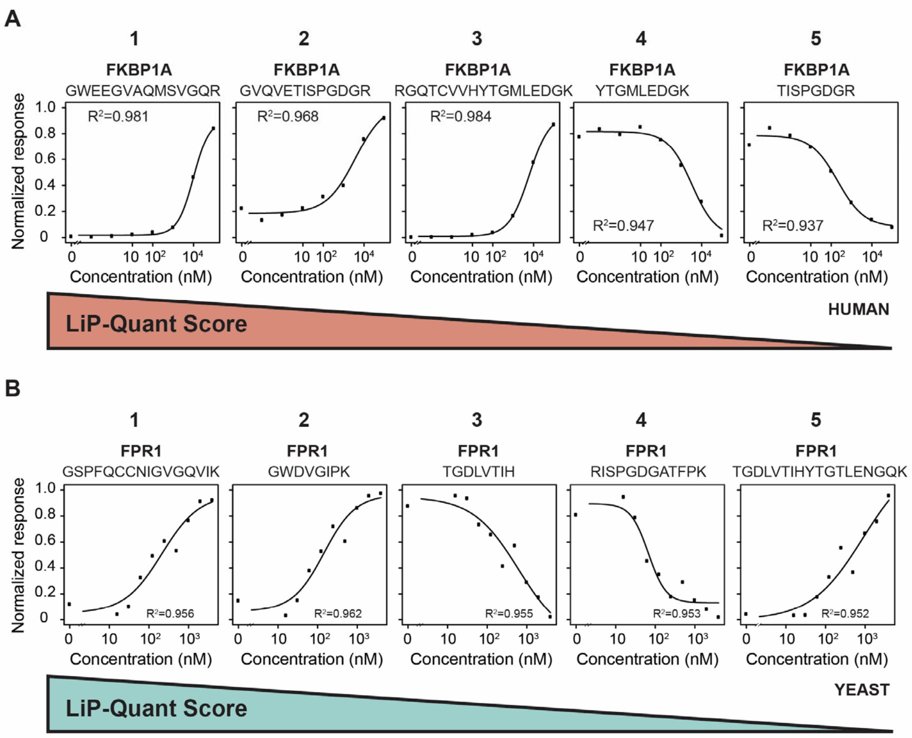

C

FPR1

LiP-Quant peptide	R^2	Score	Rank
**GSPFQCCNIGVGQVIK**	0.956	3.228	**1**
GWDVGIPK	0.962	3.111	2
TGDLVTIH	0.955	3.070	3
RISPGDGATFPK	0.953	2.974	4
TGDLVTIHYTGTLENGQK	0.952	2.771	5

D

Other protein targets

LiP-Quant peptide (protein)	R^2	Score	Rank
AHLVAIEKPECTGQR (**ARI1**)	0.969	2.711	6
GDLVITEESWNK (**ARI1**)	0.952	2.581	8
APIVAYAELIAAR (**SYEC**)	0.937	2.510	10

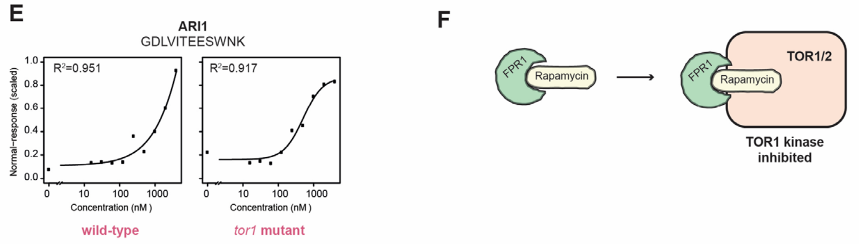

Supplementary Figure 4: Consistency of LiP-Quant across compounds and species.

A: Top 5 ranked LiP-Quant peptides by LiP-Quant score generated in an experiment using FK506. The compound targets the same protein (FKBP1A) in the same binding site as rapamycin and generates the same peptides. Numbers atop the graphs represent each peptide's LiP score rank. In both experiments the LiP-Quant peptides show similar regulation, with peptide intensity changes in the same direction (log2 fold change) upon drug binding. **B:** Dose-response curves showing relative intensities of LiP-Quant peptides obtained from *S. cerevisiae* lysates over a rapamycin concentration range (0 nM to 2000 nM). Curves of the top 5 LiP-Quant peptides ranked by LiP-Quant score are shown, all of which are from FPR1, the expected direct target for this drug in yeast cells. **C:** Table of dose response correlation (R^2) and LiP score for peptides from B. **D:** LiP-Quant peptides ranking in positions 6, 8, 10 of the LiP-Quant experiment from B. **E:** Dose-response curves of the relative peptide intensity of GDLVITEESWNK, which maps to ARI1, over a concentration range of rapamycin lysates of wild type or *tor1-fpr1* mutant yeast. **F:** Model of TOR1 kinase inactivation through the binding of rapamycin to FPR1.

A

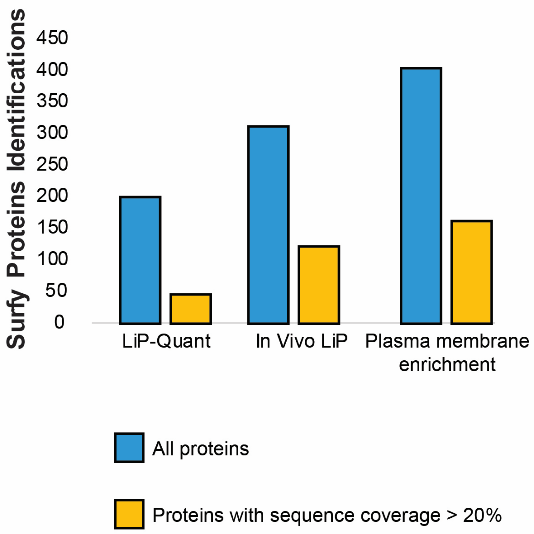

B

ATP1A1

LiP-Quant peptide	R2	EC50	Rank
AVAGDASESALLK	0.972	16.39	1
RAVAGDASESALLK	0.948	47.39	33
GGDRIPADLR	0.915	51.72	158

C

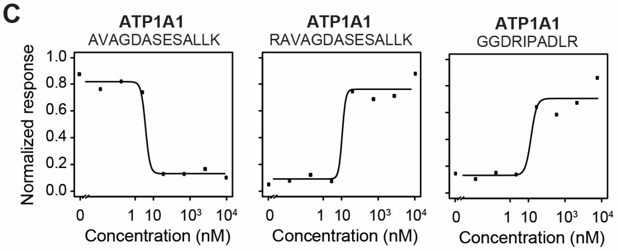

Supplementary Figure 5: LiP-Quant and plasma membrane protein targets

A: Total membrane protein identifications (blue) and those with protein coverage above 20% (yellow) in HeLa cells based upon SURFY classification [24] from a representative LiP-Quant experiment, *in vivo* LiP (Figure 1A) and the average of four membrane enrichment experiments. **B:** Top 3 ranking peptides for ATP1A1 (the known target of proscillaridin A) in a LiP-Quant experiment. Correlation coefficients (R^2) and extrapolated EC50 values are reported for each peptide. **C:** Dose-response curves showing relative intensities of LiP-Quant peptides for ATP1A1 from B.

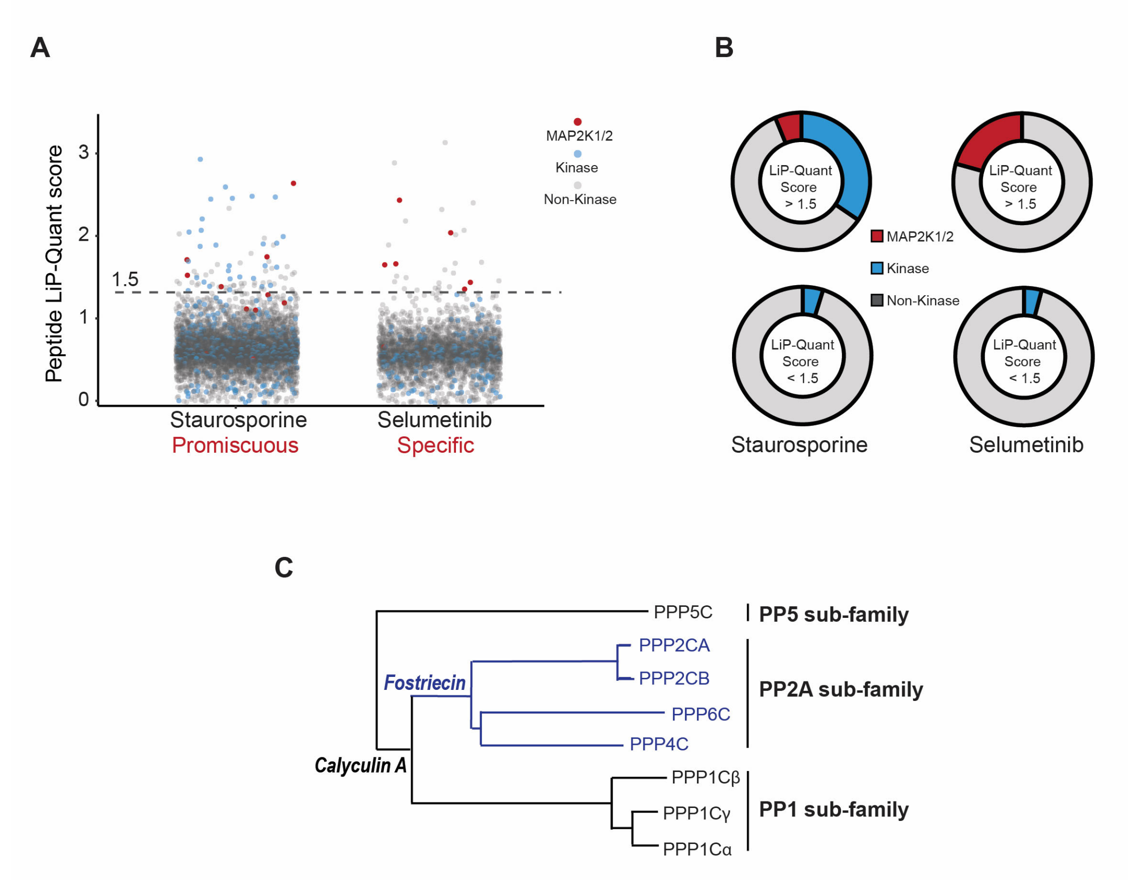

Supplementary Figure 6: LiP-Quant is a sensitive method for drug targets of different specificity

A: LiP-Quant captures druggable kinase targets with different specificity: Jitter-plots showing the distribution of LiP-Quant scores in LiP-Quant experiments done with the indicated kinase inhibitors. The blue dots show peptides assigned a score from any kinase, while gray dots show peptides assigned a LiP-Quant score from all other classes of proteins. LiP-Quant peptides of MAPK proteins are shown in red. The LiP-Quant score cut-off for expected targets is shown with a dashed line. **B:** Radar plots showing the proportion of LiP-Quant peptides corresponding to protein kinases among peptides with a LiP-Quant score higher or lower than the threshold score of 1.5. **C:** Phylogenetic tree (Clustal X neighbor joining tree) of the protein phosphatase (PP) family including subfamilies. The colors show the known sub-family selectivity of the compounds calyculin A and fostriecin.

A

MAP2K1

LiP-Quant peptide	R2	EC50	Rank
VSIAVIK	0.910	100.96	3
VSHKPSGLVMAR	0.858	48.50	8
LCDFGVSGQLIDSMANSF VGTR	0.786	53.25	13

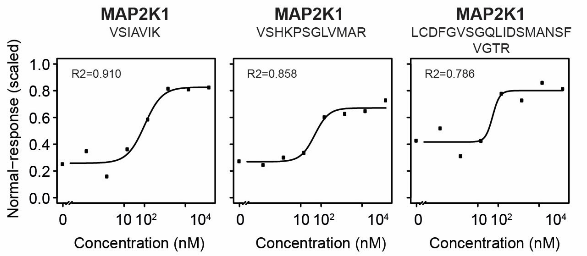

B

PP2A

LiP-Quant peptide	R2	EC50	Rank
YSFLQFDPAPR	0.990	18.22	1
NVVTIFSAPNYCYR	0.984	19.28	2
AHQLVMEGYNWCHDR	0.979	11.38	3

PP1A

LiP-Quant peptide	R2	EC50	Rank
IKYPENFFLLR	0.951	663.0	4
EIFLSQPILLELEAPLK	0.946	55.47	5
IYGFYDECKR	0.953	63.26	7

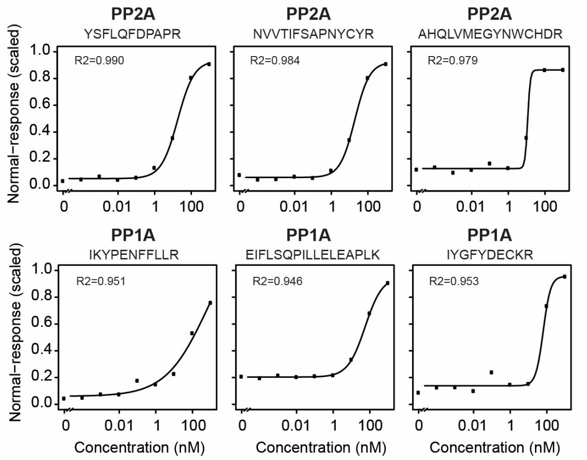

Supplementary Figure 7: Quantitative measurements of drug binding in cell lysates

A: Top 3 ranking LiP-Quant peptides for MAPK2K1 in a LiP-Quant experiment with selumenitib with their relative peptide intensities plotted in response to drug dose. Correlation coefficients (R^2) and extrapolated EC50 values are reported for each peptide. **B:** Top 3 ranking LiP-Quant peptides for PP2A (top panel) and PP1A (bottom panel) in a LiP-Quant experiment with calyculin A with their relative peptide intensities plotted in response to drug dose. Correlation coefficients (R^2) and extrapolated EC50 values are reported for each peptide.

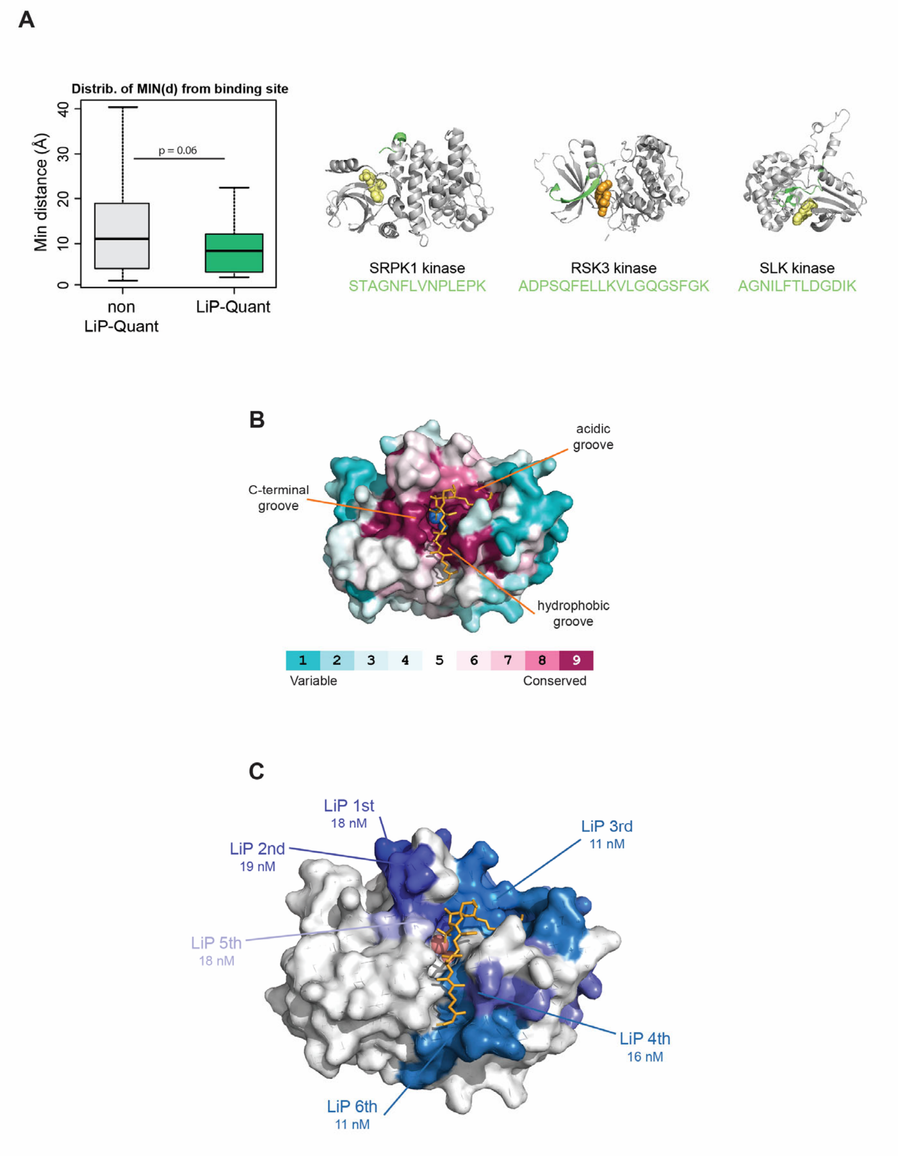

Supplementary Figure 8: Peptide-level structural analysis by LiP-Quant

A: Distribution of minimal distances from the drug binding site calculated for LiP-Quant and non-LiP-Quant peptides identified in the experiment with staurosporine. Holocomplexes of kinases bound to the kinase inhibitors or ATP were used for this analysis. The median distance from the drug binding site is 8.3 Å for LiP-Quant peptides and 11.4 Å for non-LiP-Quant peptides. Representative holocomplex structures of SRPK1, RSK3 and SLK kinases with their drug or ATP ligands are shown. LiP-Quant peptides are colored in green; drug ligand in yellow, ATP in orange. In the box plots the central line defines the median, the bounds of box the first and third quartile, the whiskers are the minimal and maximum values. **B**: Structural model of calyculin A bound to the PP1-gamma catalytic subunit (PDBID: 1it6). The surface has been colored in pink or blue according to amino acid conservation. calyculin A (represented with orange sticks) occupies the hydrophobic groove and the acidic groove on the molecular surface and adopts an extended conformation on the surface. **C**: Surface representation of a structural model of calyculin A bound to PP2A with the surfaces corresponding to the top 6 ranking peptides by LiP-Quant score of PP2A (calyculin A experiment) colored with different tones of blue. Since the catalytic subunits of the PP family are highly structurally similar, the

PP1-gamma subunit has been used as a model of the complex between PP2A and the drug after aligning the PP2A peptides to their homologs of PP1-gamma (PDBID: 1it6).

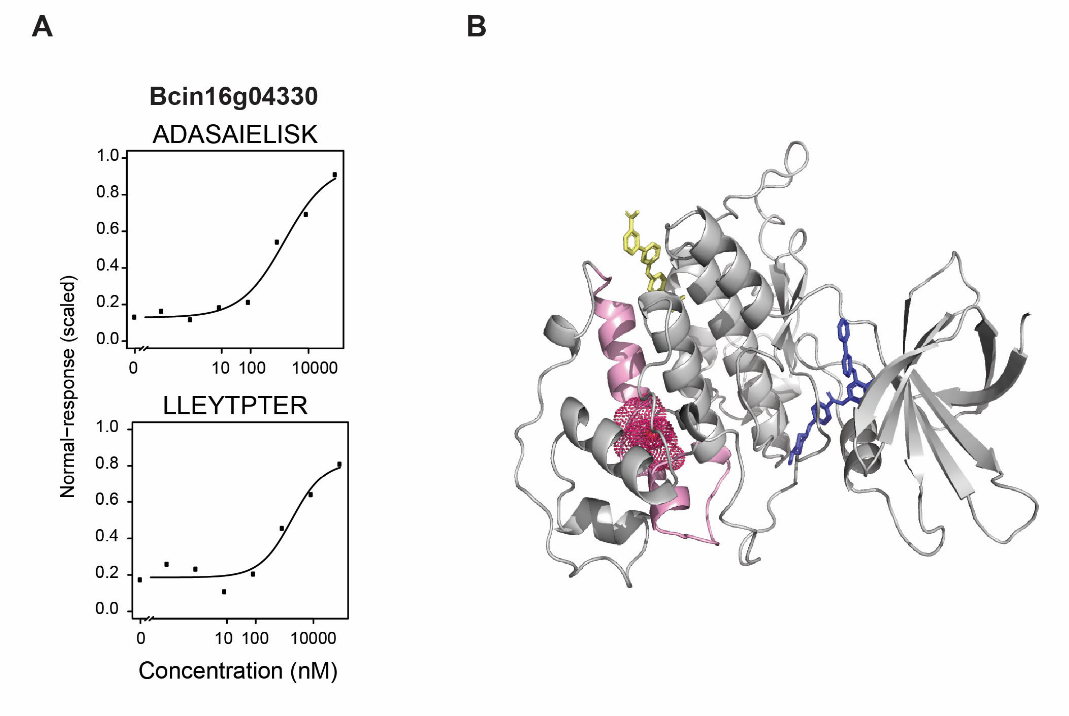

Supplementary Figure 9: Identification of a secondary kinase target and its binding site of a novel fungicide

A: Dose-response curves showing relative intensities of the top two ranking LiP-Quant peptides for Bcin16g04330 (see Figure 4C). The extrapolated average EC50 for this protein is 1.6 μM. **B:** Structure of Bcin16g04330, a serine/threonine kinase, predicted by homology modelling (template PDBID: 3k5v), showing the position of the top ranking LiP-Quant peptides for this protein (pink) mapping outside the catalytic site (shown with the ligand from PDBID: 3k5v bound to it). The ligand shown in yellow shows the position of a common allosteric site of this family of kinases [33].

Supplementary Note 1: Synthetic procedures for the synthesis and characterization of BAYE-004

General

Unless otherwise noted, all reactants or reagents including dry solvents were obtained from commercial suppliers and used as received. Unless otherwise noted, all reactions were performed with dry solvents under an atmosphere of argon in dried glassware using standard vacuum-line techniques. All work-up and purification procedures were carried out with reagent-grade solvents in air. Analytical thin-layer chromatography (TLC) was performed using E. Merck silica gel 60 F254 precoated plates (0.25 mm). The developed chromatogram was analyzed by UV lamp (254 nm). Flash column chromatography was performed with E. Merck silica gel 60 (230–400 mesh) or ISCO Combiflash Rf & Rf200® instrument equipped with Chromabond Flash cartridges. Nuclear magnetic resonance (NMR) spectra were recorded on a Bruker AV400 spectrometer. Chemical shifts for 1H NMR are expressed in parts per million (ppm) relative to tetramethylsilane (δ 0.00 ppm). Data are reported as follows: chemical shift, multiplicity (s = singlet, d = doublet, dd = doublet of doublets, ddd = doublet of doublets of doublets, t = triplet, m = multiplet, br = broad signal), coupling constant (Hz), and integration.

Compound Synthesis

1-(4-fluorophenyl)-6-hydroxyhexane-1,3-dione

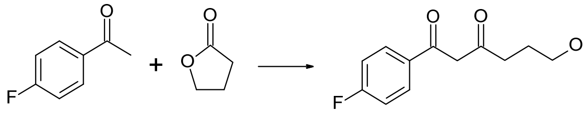

Starting with a mixture of sodium hydride (60%, 20g, 0.5 mol) in 1 L dry diethyl ether, 2 ml of ethanol was added at 0°C under argon followed by γ-butyrolactone (18.8 g, 0.21 mol). Next, a solution of 4-fluorophenyl acetophenone (29.0 g , 0.21 mol) in 100 mL diethyl ether was added slowly at 0°C and the resulting suspension was allowed to warm slowly to room temperature (RT), followed by 72 h of stirring at RT. 20 ml of ethanol was added to the resulting reddish-brown suspension, followed by 200 mL ammonium chloride solution. The organic phase was separated and the aqueous phase was extracted with ethyl acetate. The combined extracts were dried and the volatiles removed under vacuum. Next, the oily residue was triturated with hexane and 1-(4-fluorophenyl)-6-hydroxyhexane-1,3-dione was obtained as solid (28g, 81%). 1H-NMR (400MHZ, CD3CN): δ = 8.09-7.98 (m, 2H), 7.28-7.22 (m, 2H), 6.37 (s, 1H), 3.59-3.49 (m, 2H), 2.53 (t, 2H), 1.86-1.70 (m, 2H) ppm

3-[3-(4-fluorophenyl)-1H-pyrazol-5-yl]propan-1-ol

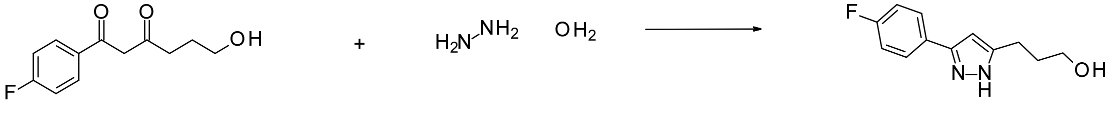

Beginning with a solution of 1-(4-fluorophenyl)-6-hydroxyhexane-1,3-dione (38g, 0.17 mol) in 150 mL ethanol, hydrazine hydrate (17.05g, 0.34 mol) was added slowly over a 30 minute period (exothermic up to 35°C). The resulting solution was stirred for 1 h at RT and then the mixture was added to aqueous ammonium chloride. Extraction with ethyl acetate and evaporation of the organic phase resulted in crude product. The product was purified by trituration with 2 volumes (200 mL) of n-hexane and the product was obtained as solid (29g, 77%). 1H-NMR (400 MHz, de-DMSO): δ = 12.56 (s, 1H), 7.80-7.76 (m, 2H), 7.22-7.18 (m,

2H), 6.45 (s, 1H), 4.52 (m, 1H), 3.47-3.32 (q, 2H), 2.64 (m, 2H), 1.80-1.70 (m, 2H) ppm logP (pH 2.7): 1.51MS (ESI): 221.1 ([M+H]+)

3-[3-(4-fluorophenyl)-1H-pyrazol-5-yl]propyl methanesulfonate

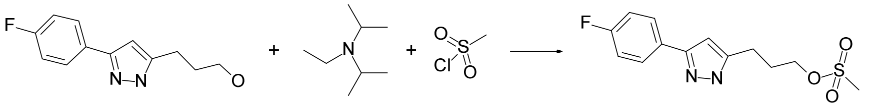

To a solution of 3-[3-(4-fluorophenyl)-1H-pyrazol-5-yl]propan-1-ol (28.6g, 0.13mol) in 250 mL dichloromethane, diisopropylethylamine (25.2g, 0.195mol, 1.5eq), and methanesulfonyl chloride (17.8g, 0.156mol, 1.2eq) were added at 10°C. The mixture was stirred for 30 mins at 10°C and then allowed to warm to ambient temperature over an additional 30 mins. 50 mL of aqueous Na_2CO_3 solution was then added and the phases were separated. After drying, 52g of the product was obtained. logP (pH 2.7): 2.00MS (ESI): 299.1 ([M+H]+)*

*The crude product consists of a mixture of mesylate and chloride (~ 7:3 ratio) by LC-MS and was used without further purification in the next step.

2-(4-fluorophenyl)-5,6-dihydro-4H-pyrrolo [1,2-b] pyrazole

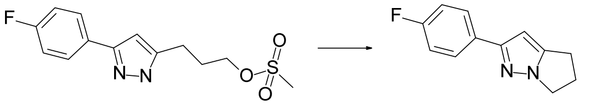

Sodium iodide (1.84g, 12.2mmol) was added at 10°C to a solution of crude 3-[3-(4-fluorophenyl)-1H-pyrazol-5-yl]propyl methanesulfonate (52g, 0.122mol), in 300 mL N,N'-dimethylformamide. Next, sodium hydride (60%, 5.4g, 0.134mol) was added portion-wise and the mixture was stirred for 30 mins at 10°C followed by 1 h at room temperature. To the resulting brown suspension saturated ammonium chloride was added and the mixture was extracted with ethyl acetate. The extracts were dried and evaporated under vacuum. The obtained crude product was purified by trituration with 50 mL water plus 50 mL n-hexane. After drying of the solid in vacuo, 22.9g (90%) of the product was obtained was used without further purification in the next step. 1H-NMR (400 MHz, CD3CN): δ = 7.80-7.75 (m, 2H), 7.14-7.08 (m, 2H), 6.29 (s, 1H), 4.12 (t, 2H, J = 7 Hz), 2.90 (t, 2H, J = 7 Hz), 2.59 (m, 2H) ppm. logP (pH 2.7): 2.39MS (ESI): 203.1 ([M+H]+)

3-bromo-2-(4-fluorophenyl)-5,6-dihydro-4H-pyrrolo[1,2-b]pyrazole

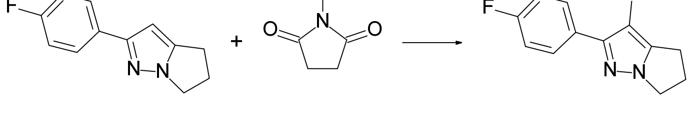

To a solution of 2-(4-fluorophenyl)-5,6-dihydro-4H-pyrrolo[l,2-b]pyrazole (44.5g, 0.22mol) (in 600 mL chloroform), N-bromosuccinimide (117g, 0.66mol) was added at 0°C. The mixture was then stirred for 17 h at RT. Thereafter, the mixture was diluted with water and extracted with dichloromethane. The combined organic phases were dried and evaporated. The obtained crude material was purified by column chromatography on silica (6cm x 40cm, eluent dichloromethane). The obtained product was further purified by trituration with an

MTBE-hexane mixture and 38.2g (61%) of the product was obtained as solid. 1H-NMR (400 MHz, CD3CN): δ = 7.89-7.84 (m, 2H), 7.20-7.14 (m, 2H), 4.16 (t, 2H, J = 7 Hz), 2.87 (m, 2H), 2.64-2.55 (m, 2H) ppm. logP (pH 7): 3.21MS (ESI): 283.0 ([M+H]+)

4-[2-(4-fluorophenyl)-5,6-dihydro-4H-pyrrolo[1,2-b]pyrazol-3-yl]pyridin-2-amine

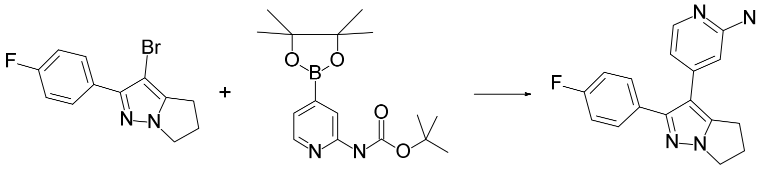

3-bromo-2-(4-fluorophenyl)-5,6-dihydro-4H-pyrrolo[1,2-b]pyrazole (700mg, 2.5mmol) and tert-butyl [4-(4,4,5,5-tetramethyl-l,3,2-dioxaborolan-2-yl)pyridin-2-yl]carbamate (727mg, 2.27mmol, 1.1 eq) were dissolved in 10 mL of 1,4-dioxan. Next, Bis(tricyclohexylphosphine)palladium(II)-dichloride (168mg, 0.22mmol, 0.1 eq) and 5.4mL sodium carbonate solution (2M) was added to this solution. The reaction mixture was flushed with argon for 5 mins and then sealed. Next, the mixture was heated for 12 mins at 150°C in a microwave (Biotage). After cooling, insoluble components were filtered off over Celite and the residue was washed with 1,4-dioxan. The organic phase was evaporated and the crude product was purified by column chromatography over silica gel using dichloromethane / methanol (95:5) as eluent. After evaporation of the solvents 530mg (72%) of 4-[2-(4-fluorophenyl)-5,6-dihydro-4H-pyrrolo[1,2-b]pyrazol-3-yl]pyridin-2-amine was obtained as a colourless solid. 1H-NMR (400 MHz, CD3CN): δ = 7.84-7.82 (d, 1H, J = 5.3 Hz), 7.49-7.44 (m, 2H), 7.11-7.05 (m, 2H), 6.40-6.38 (dd, 1H, J = 5.3, 1.5 Hz), 6.34 (s, 1H), 4.75 (s, 2H), 4.14 (t, 2H, J = 7 Hz), 3.00 (t, 2H, J = 7 Hz), 2.66-2.59 (m, 2H) ppm logP (pH 2.7): 1.02MS (ESI): 295.2 ([M+H]+)

N-[4-[2-(4-fluorophenyl)-5,6-dihydro-4H-pyrrolo[1,2-b]pyrazol-3-yl]-2-pyridyl]propanamide

[BAYE-004]

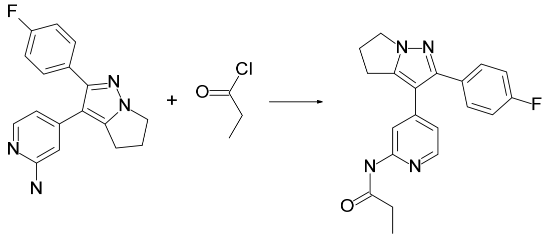

A solution of propanoyl chloride (74mg, 0.8mmol) was added to a solution of 4-[2-(4-fluorophenyl)-5,6-dihydro-4H-pyrrolo[1,2-b]pyrazol-3-yl]pyridin-2-amine (117mg, 0.4 mmol) and Huenigs base (62mg, 0.48 mmol) in 2 mL tetrahydrofuran. The resulting mixture was stirred for 16h at 25°C then 1 mL of a 4M NH_3 solution in methanol was added. The mixture was stirred for a further 16h. After evaporation of the volatile solvents the crude product was purified by chromatography and the product was obtained as solid (55mg, 39%). 1H-NMR (400 MHz, d3-CD3CN): δ = 8.50 (s, 1H, br), 8.10-8.06 (m, 2H), 7.48-7.44 (m, 2H), 7.11-7.06 (m, 2H), 6.80 (dd, 1H, J = 5.7, 1.5 Hz), 4.16 (t, 2H, J = 7.3 Hz), 3.05 (t, 2H, J = 7 Hz), 2.66 (m, 2H), 2.38 (q, 2H), 1.10 (t, 3H) ppm. logP (HCOOH): 1.67, MS (ESI): 351.5 ([M+H]+)

A:

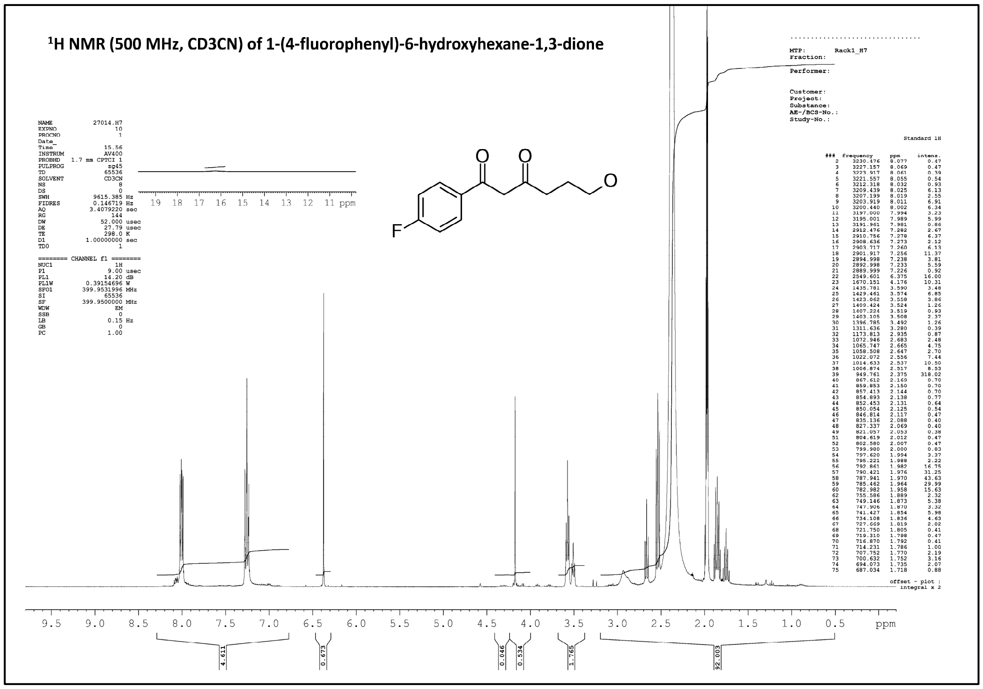
1H NMR (500 MHz, CD3CN) of 1-(4-fluorophenyl)-6-hydroxyhexane-1,3-dione

B:

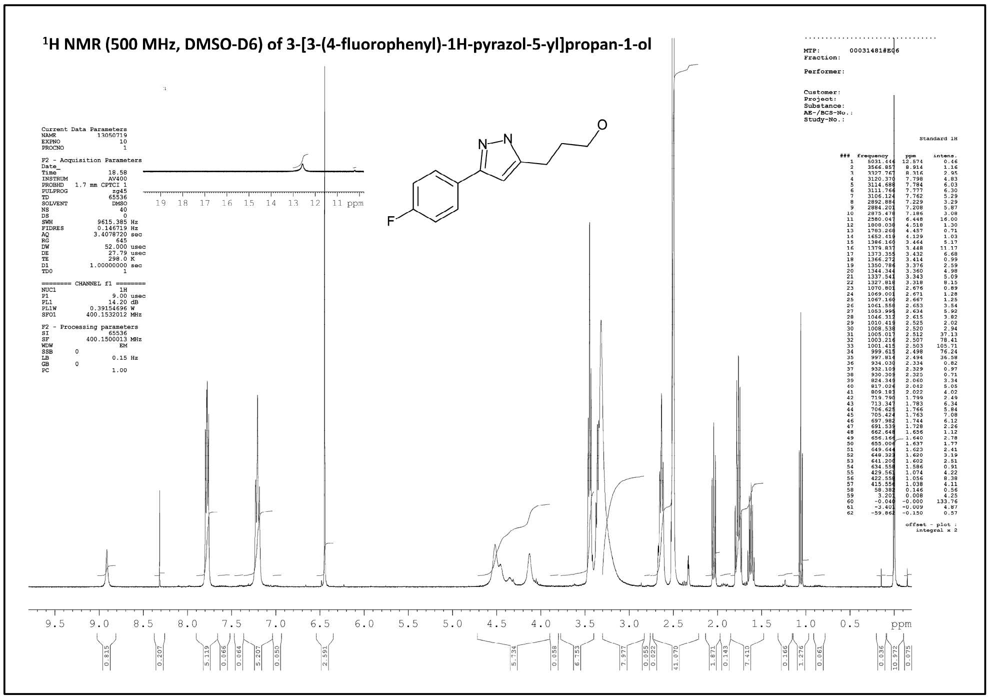
1H NMR (500 MHz, DMSO-D6) of 3-[3-(4-fluorophenyl)-1H-pyrazol-5-yl]propan-1-ol

C:

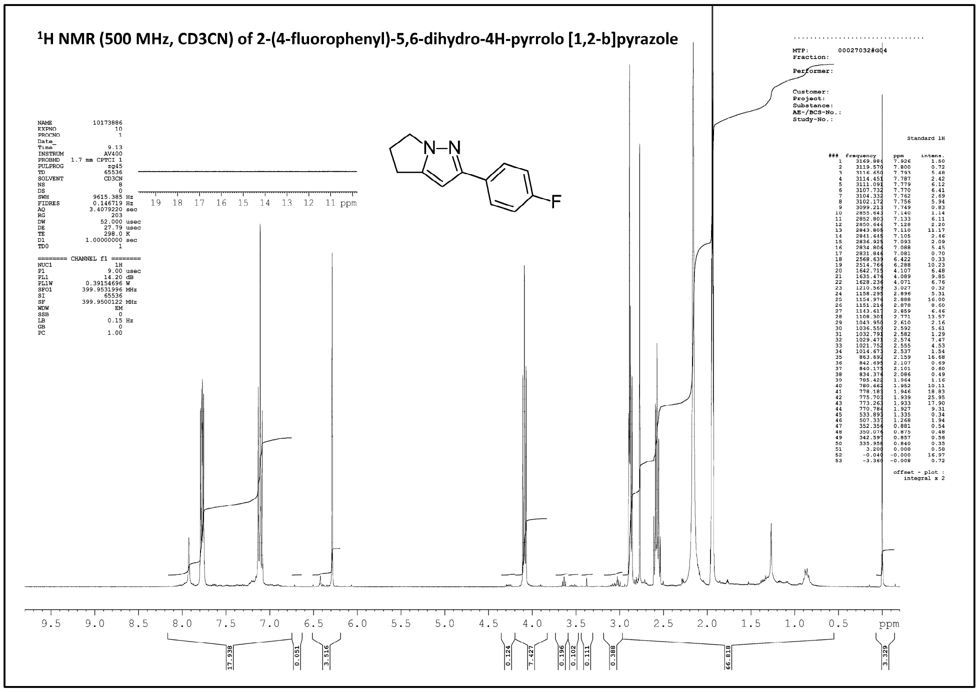

1H NMR (500 MHz, CD3CN) of 2-(4-fluorophenyl)-5,6-dihydro-4H-pyrrolo [1,2-b]pyrazole

D:

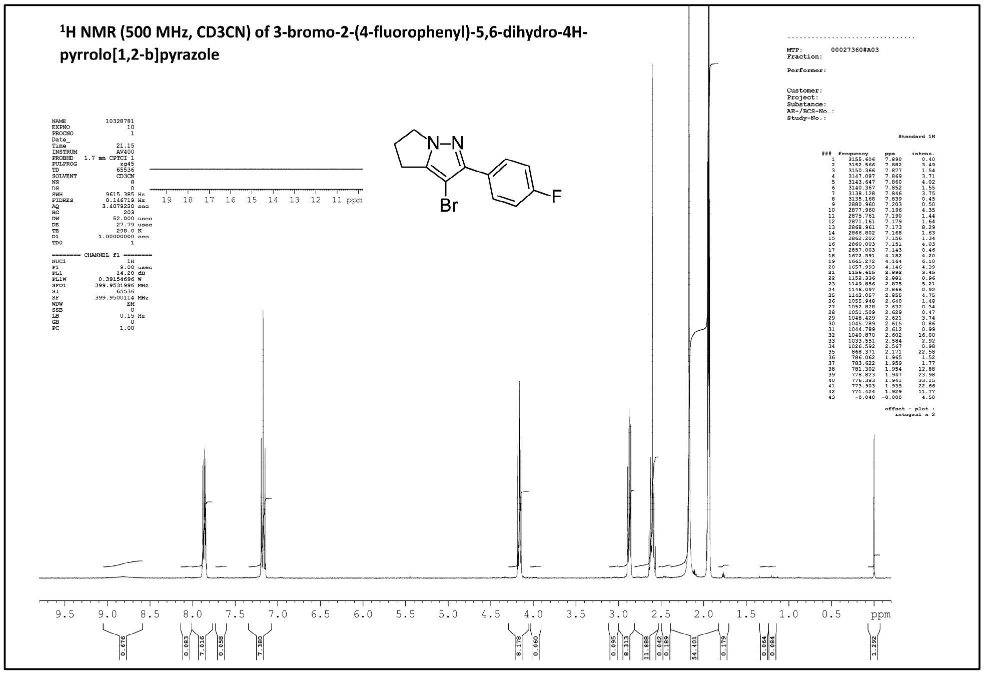

1H NMR (500 MHz, CD3CN) of 3-bromo-2-(4-fluorophenyl)-5,6-dihydro-4H-pyrrolo[1,2-b]pyrazole

E:

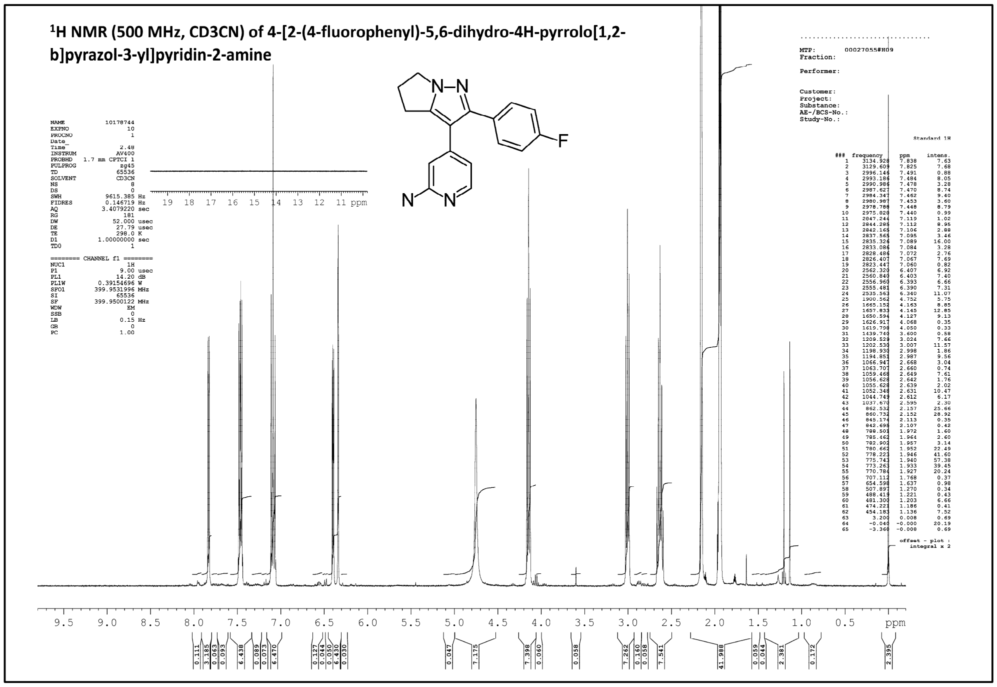

F:

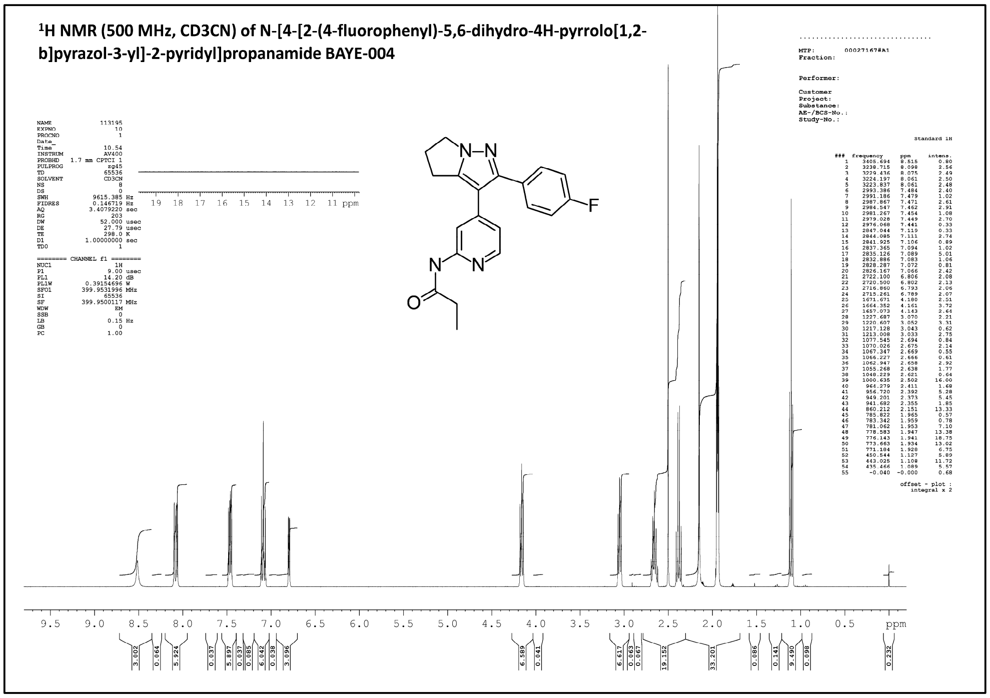

Supplementary Figure 10: NMR spectra of compounds throughout synthesis of BAYE-004

A: 1H NMR (500 MHz, d3-CD3CN) of 1-(4-fluorophenyl)-6-hydroxyhexane-1,3-dione. **B:** 1H NMR (500 MHz, DMSO-D6) of 3-[3-(4-fluorophenyl)-1H-pyrazol-5-yl]propan-1-ol. **C:** 1H NMR (500 MHz, CD3CN) of 2-(4-fluorophenyl)-5,6-dihydro-4H-pyrrolo [1,2-b] pyrazole. **D:** 1H NMR

(500 MHz, CD3CN) of 3-bromo-2-(4-fluorophenyl)-5,6-dihydro-4H-pyrrolo[1,2-b]pyrazole. **E:** 1H NMR (500 MHz, CD3CN) of 4-[2-(4-fluorophenyl)-5,6-dihydro-4H-pyrrolo[1,2-b]pyrazol-3-yl]pyridin-2-amine. **F:** 1H NMR (500 MHz, CD3CN) of N-[4-[2-(4-fluorophenyl)-5,6-dihydro-4H-pyrrolo[1,2-b]pyrazol-3-yl]-2-pyridyl]propenamide [BAYE-004].

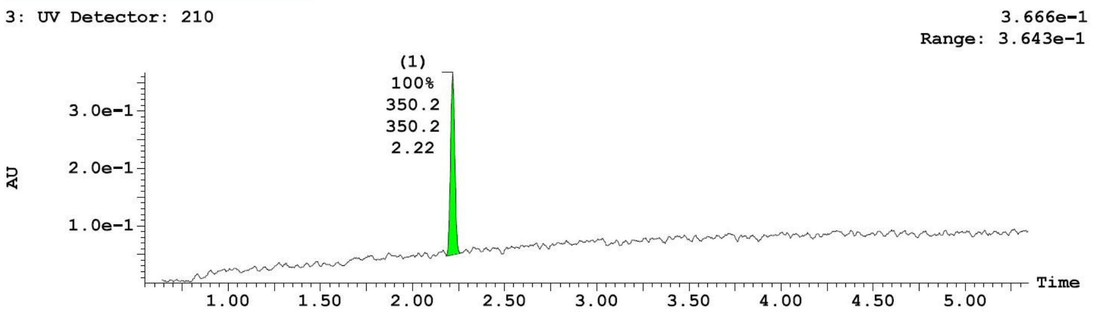

Peak Number	Compound	Time	AreaAbs	Area %Total	Width	Height	Mass Found	RT LogP
1	Found	2.22	9e+003	100.00	0	3e+005	350.15, 350.15	1.67

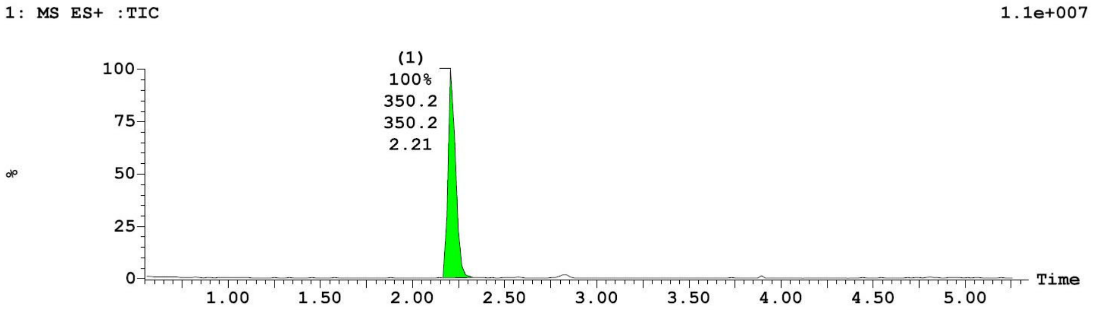

Peak Number	Compound	Time	AreaAbs	Area %Total	Width	Height	Mass Found	RT LogP
1	Found	2.21	5e+005	100.00	0	1e+007	350.15, 350.15	1.66

Supplementary Figure 11: LC-MS of BAYE-004 compound
