# Supplementary material for: A machine learning-based chemoproteomic approach to identify drug targets and binding sites in complex proteomes
Source: Nat Commun. 2020 Aug 21;11:4200. doi: 10.1038/s41467-020-18071-x (PMC7442650; doi:10.1038/s41467-020-18071-x)
Supplement: Supplementary file 3 — Description of Additional Supplementary Files [file 41467_2020_18071_MOESM3_ESM.pdf]

Description of Additional Supplementary Files

Title: Supplementary Data 1.
Description: LiP-SMap experiment of HeLa cell lysates treated with rapamycin at a concentration of 2 µM.

Title: Supplementary Data 2.
Description: LiP-SMap experiment of HeLa live cell cultures treated with rapamycin at a concentration of 2 µM.

Title: Supplementary Data 3.
Description: LiP-Scores obtained after analysis with the LiP-Quant machine learning-based classifier of HeLa cell lysates treated with the following compounds: rapamycin, FK506, calyculin A, fostriecin, selumetinib, staurosporine.

Title: Supplementary Data 4.
Description: Consistency of LiP-Quant scores and differential peptide abundance intensities with rapamycin and FK506.

Title: Supplementary Data 5.
Description: LiP scores obtained after analysis with the LiP-Quant machine learning-based classifier of *S.cerevisiae* cell lysates from *wild type* and ∆*Frb1* mutant strains.

Title: Supplementary Data 6.
Description: KinHub kinase reference database (http://www.kinhub.org).

Title: Supplementary Data 7.
Description: Benchmarking of LiP-Quant and Deep-LiP-Quant with TPP parametric (Savitski, 2014) and TPP non-parametric analysis (Childs, 2019).

Title: Supplementary Data 8.
Description: Deep-LiP-Quant experiment of HeLa cell lysates treated with staurosporine and analyzed with a 4-hours LC gradient.

Title: Supplementary Data 9.
Description: LiP-Scores obtained after analysis with the LiP-Quant machine learning-based classifier of HeLa membrane-enriched fractions.

Title: Supplementary Data 10.
Description: Single concentration LiP-Quant rapamycin control experiment of HeLa cell lysates treated with mild detergent during cell lysis.

Title: Supplementary Data 11.
Description: Nuanced specificity in target binding of fostriecin and calyculin A.

Title: Supplementary Data 12.
Description: Predictions of binding sites with center of mass of high confidence LiP peptides.

Title: Supplementary Data 13.
Description: LiP-Scores obtained after analysis with the LiP-Quant machine learning-based classifier of *Botrytis cinerea* cell lysates treated with the compound BAYE-004.

Title: Supplementary Data 14.
Description: Peptide Frequency Library (PFL) that identifies proteins that are common contaminants in LiP-Quant experiments.

Title: Supplementary Data 15.
Description: Peptide ranking for calculation of drug binding sites in the LiP-Quant experiments reported in this manuscript.

Title: Supplementary Data 16.
Description: List of reagents or resources used in this manuscript.
