# Supplementary material for: A machine learning-based chemoproteomic approach to identify drug targets and binding sites in complex proteomes
Source: Nat Commun. 2020 Aug 21;11:4200. doi: 10.1038/s41467-020-18071-x (PMC7442650; doi:10.1038/s41467-020-18071-x)
Supplement: Supplementary file 20 — Reporting Summary [file 41467_2020_18071_MOESM20_ESM.pdf]

nature research

Corresponding author(s): Lukas Reiter

Last updated by author(s): Jul 9, 2020

Reporting Summary

Nature Research wishes to improve the reproducibility of the work that we publish. This form provides structure for consistency and transparency in reporting. For further information on Nature Research policies, see Authors & Referees and the Editorial Policy Checklist .

Statistics

For all statistical analyses, confirm that the following items are present in the figure legend, table legend, main text, or Methods section.

n/a	Confirmed	
☐	☒	The exact sample size (*n*) for each experimental group/condition, given as a discrete number and unit of measurement
☐	☒	A statement on whether measurements were taken from distinct samples or whether the same sample was measured repeatedly
☐	☒	The statistical test(s) used AND whether they are one- or two-sided<br>*Only common tests should be described solely by name; describe more complex techniques in the Methods section.*
☒	☐	A description of all covariates tested
☐	☒	A description of any assumptions or corrections, such as tests of normality and adjustment for multiple comparisons
☐	☒	A full description of the statistical parameters including central tendency (e.g. means) or other basic estimates (e.g. regression coefficient) AND variation (e.g. standard deviation) or associated estimates of uncertainty (e.g. confidence intervals)
☐	☒	For null hypothesis testing, the test statistic (e.g. *F*, *t*, *r*) with confidence intervals, effect sizes, degrees of freedom and *P* value noted<br>*Give P values as exact values whenever suitable.*
☒	☐	For Bayesian analysis, information on the choice of priors and Markov chain Monte Carlo settings
☒	☐	For hierarchical and complex designs, identification of the appropriate level for tests and full reporting of outcomes
☒	☐	Estimates of effect sizes (e.g. Cohen's *d*, Pearson's *r*), indicating how they were calculated

Our web collection on statistics for biologists contains articles on many of the points above.

Software and code

Policy information about availability of computer code

Data collection	Programs used for data collection are Xcalibur v4.1 (ThermoFisher Scientific).
Data analysis	The R script used for analysis has been made available at GitHub (https://github.com/RolandBruderer/LiP-Quant). Programs used for data analysis are R v3.6.0 and 'drc' package (www.r-project.org), Microsoft Excel, PyMol v2.1 (Schroedinger), Proteome Discoverer v2.2 (thermoFisher Scientific) and Spectronaut Pulsar X and SpectroMine 2.0 (Biognosys AG).

For manuscripts utilizing custom algorithms or software that are central to the research but not yet described in published literature, software must be made available to editors/reviewers. We strongly encourage code deposition in a community repository (e.g. GitHub). See the Nature Research guidelines for submitting code & software for further information.

Data

Policy information about availability of data

All manuscripts must include a data availability statement. This statement should provide the following information, where applicable:

- Accession codes, unique identifiers, or web links for publicly available datasets
- A list of figures that have associated raw data
- A description of any restrictions on data availability

All mass spectrometry proteomics data have been deposited at ProteomeXchange Consortium via the PRIDE partner repository with the dataset identifiers PXD018204 (Username: reviewer49707@ebi.ac.uk, Password: gX2TmqyV) and PXD015446 (Username: reviewer72396ebi.ac.uk, Password: Y3Rb0rRh). The authors declare that all additional data supporting the findings of this study are available within the paper and its supplementary information files.

Source data are provided with this paper. The source data underlying figures 1a, 2, 4b,d,f, S2b,c, S3, S4b,e, S6a,b and S8a are provided as a source data zip file. Source data underlying figures 1d, 2c,d, 4c, S4a,b,e, S5c, S7a,b and S9a are included in the PRIDE repository with the dataset identifier PXD019902 (Username: reviewer40799@ebi.ac.uk, Password: 67zkGtcL). All other relevant data are available from the corresponding authors on request.

UniProt fasta databases for organisms were accessed on January 1st, 2018 via the UniProt databases download page (https://www.uniprot.org/downloads). Protein structures for E2AK2 kinase (PDBID: 2a19), MAPK2K1 (PDBID: 4u7z), K66A3 kinase (PDBID: 4nus), FKBP1A (FK506) (PDBID: 1fkj), FKBP1A (rapamycin) (PDBID: 2dg3) and PP2A (PDBID: 1it6) were downloaded in 2019 from the Protein Data Bank website (https://www.rcsb.org/pdb).

Field-specific reporting

Please select the one below that is the best fit for your research. If you are not sure, read the appropriate sections before making your selection.

[x] Life sciences [ ] Behavioural & social sciences [ ] Ecological, evolutionary & environmental sciences

For a reference copy of the document with all sections, see nature.com/documents/nr-reporting-summary-flat.pdf

Life sciences study design

All studies must disclose on these points even when the disclosure is negative.

Sample size
n = 4 was used for all studies except for the in vivo HeLa experiment (Figure 1a) where n = 3 was used. Sample sizes were based upon previous similar experiments (LiP-SMap) and selected to provide sufficient power for proper statistical analysis (t-test and pearson correlations).

Data exclusions
No data was excluded from analysis.

Replication
A single concentration positive control (rapamycin) was run with every experiment to confirm method reproducibility. In every experiment this replicate group was successful (i.e. the target of rapamycin, FKBP1A, was identified based upon q-value ranking). The live-cell treated single concentration rapamycin experiment was run twice independently (Fig 1a). All LiP-Quant experiments were run independently twice (with similar results) with the expception of proscillaridin A, which was run once. The rapamycin lysate detergent test experiment (supp data 10) was run once.

Randomization
Randomization was not relevant during benchwork as each experiment used a homogenous cell population. Samples were block randomized by replicate group during mass spec acquisition.

Blinding
Blinding was not relevant to this study as there are no humans or animals involved.

Reporting for specific materials, systems and methods

We require information from authors about some types of materials, experimental systems and methods used in many studies. Here, indicate whether each material, system or method listed is relevant to your study. If you are not sure if a list item applies to your research, read the appropriate section before selecting a response.

Materials & experimental systems

n/a	Involved in the study	
[ ]	[x]	Antibodies
[ ]	[x]	Eukaryotic cell lines
[x]	[ ]	Palaeontology
[x]	[ ]	Animals and other organisms
[x]	[ ]	Human research participants
[x]	[ ]	Clinical data

Methods

n/a	Involved in the study	
[x]	[ ]	ChIP-seq
[x]	[ ]	Flow cytometry
[x]	[ ]	MRI-based neuroimaging

Antibodies

Antibodies used
Europium-labeled DNA topoisomerase 2-alpha(Thr1342) antibody (PerkinElmer) and anti-polyhistidine-peroxidase antibody (clone HIS-1) (Sigma-Aldrich) were used.

Validation
Validation was not carried out in-house as antibodies are confirmed in publications by the manufacturers. Further, they behaved as expected in assays (i.e. detected the proper protein weight and gave signal/no signal in positive/negative control replicates).

Eukaryotic cell lines

Policy information about cell lines

Cell line source(s)
Live HeLa cells were purchased from Sigma-Aldrich. Frozen pellets were purchased from Ipracell (Belgium). S.cerevisiae strains were a gift from the Haering lab (EMBL Heidelberg), originally obtained from Euroscarf. Botrytis cinerea strains are in house strains of Bayer SAS (originally obtained from Buttner et al).

Authentication	Certificate of authentication/analysis provided by Sigma (STR profiling). Frozen HeLa pellets were not authenticated. S.cervisiae and botrytis strains are previously published or cloned/tested in-house.
Mycoplasma contamination	Cells were tested for mycoplasma (PCR) prior to shipping by Sigma and Ipracell and tested negative.
Commonly misidentified lines (See ICLAC register)	None.
