# Supplementary figures and images for: A machine learning-based chemoproteomic approach to identify drug targets and binding sites in complex proteomes
Source: Nat Commun. 2020 Aug 21;11:4200. doi: 10.1038/s41467-020-18071-x (PMC7442650; doi:10.1038/s41467-020-18071-x)

Figure - 4D

Source data western blot (anti-polyhistidine-peroxidase antibody, 1:2000, clone HIS-1, Sigma, A7058)

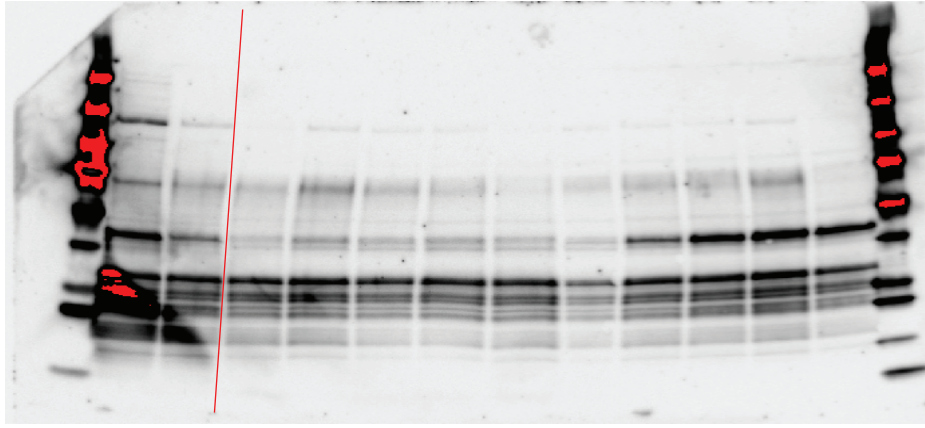

Supplement: Supplementary file 21 — Source Data [file 41467_2020_18071_MOESM21_ESM.zip › Source Data/Fig 4D Source Blot.pdf]
